# Molecular Docking Using Chimera and Autodock Vina Software for Nonbioinformaticians

**DOI:** 10.2196/14232

**Published:** 2020-06-19

**Authors:** Sania Safdar Butt, Yasmin Badshah, Maria Shabbir, Mehak Rafiq

**Affiliations:** 1 Atta Ur Rahman School of Applied Biosciences National University of Sciences and Technology Islamabad Pakistan; 2 Research Centre for Modelling and Simulation National University of Sciences and Technology Islamabad Pakistan

**Keywords:** virtual screening, molecular docking, Autodock Vina, UCSF Chimera, nonbioinformaticians, binding affinity, protein database, computer-aided docking, free offline docking, drug discovery

## Abstract

In the field of drug discovery, many methods of molecular modeling have been employed to study complex biological and chemical systems. Experimental strategies are integrated with computational approaches for the identification, characterization, and development of novel drugs and compounds. In modern drug designing, molecular docking is an approach that explores the confirmation of a ligand within the binding site of a macromolecule. To date, many software and tools for docking have been employed. AutoDock Vina (in UCSF [University of California, San Francisco] Chimera) is one of the computationally fastest and most accurate software employed in docking. In this paper, a sequential demonstration of molecular docking of the ligand fisetin with the target protein Akt has been provided, using AutoDock Vina in UCSF Chimera 1.12. The first step involves target protein ID retrieval from the protein database, the second step involves visualization of the protein structure in UCSF Chimera, the third step involves preparation of the target protein for docking, the fourth step involves preparation of the ligand for docking, the fifth step involves docking of the ligand and the target protein as Mol.2 files in Chimera by using AutoDock Vina, and the final step involves interpretation and analysis of the docking results. By following the guidelines and steps outlined in this paper, researchers with no previous background in bioinformatics research can perform computational docking in an easier and more user-friendly manner.

## Introduction

In the modern era of pharmaceutical research, many methods of molecular modeling have been employed to study complex chemical and biological systems in a variety of programs of drug discovery. It is very important to integrate experimental strategies into computational approaches in the identification, characterization, and development of novel and propitious compounds. Molecular docking is an approach used extensively in modern drug designing and development; it explores the conformations of ligands within the macromolecular target binding site, providing an estimation of receptor-ligand binding free energy for all different conformations. Small molecular compounds (ligands) are docked into the binding site of the receptor, following which the binding affinity of the complex is estimated. This constitutes a significant part of the structure-based drug design process. For a thorough understanding and estimation of the ligand/protein complex, the ability to visualize the binding interactions and geometries by using a fast and accurate protocol for docking is required [1].

To date, a variety of algorithms for docking are available, which can lead to a better understanding of the benefits and drawbacks of these methods. However, most of the free tools rely on the knowledge of the command-line interface. For biologists, this is a laborious process and hence they avoid it. The proper estimation of each method can lead to the development of plausible strategies and the origination of reproducible and relevant results.

Autodock and AutoDock Vina (The Scripps Research Institute) are some of the most widely used, free, open-source tools for molecular docking simulations [2]. AutoDock is a collection of command-line programs that can be employed to predict binding conformations of a small flexible ligand to a macromolecular target whose structure is known. This technique combines the rapid grid-based method used for energy evaluation with conformation searching and simulated annealing.

AutoDock 4 was used for molecular docking previously. The new AutoDock Vina has a more accurate binding algorithm that can speed up the rate by approximately 2 orders of magnitude as compared to AutoDock 4. In addition, AutoDock 4 has significantly improved predictions of binding mode, assessed by the training tests employed in the AutoDock 4. By the use of multithreading on the multicore machines, faster processing can be achieved from parallelism. AutoDock Vina clusters the results for the user in a transparent way and automatically calculates the grid maps.

The UCSF (University of California, San Francisco) Chimera software is used for visualization as well as analysis of density maps, 3D microscopy, molecular structures, and the associated data [3]. The challenges in the scope, size, and types of data used with the experimental cutting-edge methods are addressed by this software. It provides advanced options for high-quality rendering (reliable calculations of the molecular surface, interactive ambient occlusion, etc) and provides professional approaches to the design and distribution of the software. Chimera is a freely available software for noncommercial use and shows advances particularly in its performance, extensibility, visualization, and usability.

Chimera is segmented into major components: a core that has a role in providing visualization and basic services and extensions that have a higher-level functionality. Two major extensions of Chimera are very important: the first one is the multiscale, which can visualize the molecular assemblies of large-scale components such as the viral coats, and the second one is collaborative interface, which allows sharing of the chimera session interactively, despite being at separate locales. The other extensions of chimera include the Multalign Viewer, which shows multiple sequence alignments and the associated structures, the Movie that replays the trajectories of molecular dynamics, the Volume Viewer that is responsible for displaying and analyzing the volumetric data, and ViewDock that screens the docked ligand orientations. Chimera is available for all operating systems. It can be freely used by academic and nonprofit users.

For the purpose of this protocol, Akt and flavonoid fisetin are used. Protein kinase B, also known as Akt, is a serine/threonine-specific protein that regulates cell growth and survival [4]. In various cancers, the PI3K or Akt signaling cascade is upregulated and linked with enhanced progression and proliferation of cancer cells. Akt is an important part of signaling cascades for cell endurance and growth throughout the progression and proliferation in cancer. It controls the cell cycle, growth, and survival by indirectly altering cyclin D1 levels and directly activating inhibitors of cyclin-dependent kinases (WAF1/p21 and KIP1/p27) [5].

The plant-derived flavonoid named fisetin present in various edible natural sources is reported to possess antiproliferative potential [6]. Invasion, proliferation, and metastatic growth are inhibited significantly by the use of various concentrations of fisetin, especially in lung cancer. Current research has reported that the PI3K/Akt cascade is a direct target of fisetin in human cells, which is a hallmark for growth and survival [7].

The tools employed in Chimera are robust, simple, and interactive, and the computations involved take a few seconds. The major benefit of Chimera is that it integrates a large collection of interactive methods. These tools also play a role in the preparation of input and examination of results from more specialized, complex, and noninteractive algorithmic analysis software. Both the interactive and the noninteractive analyses are beneficial.

## Methods

### Requirements for Docking

Docking requires the following: (1) Windows 7, 8, or 10 or Mac operating system and Linux, and (2) UCSF Chimera 1.12.

### Instructions

The stepwise instructions for docking are provided below:

#### Retrieval

Retrieve the required target protein structure from the major database Protein Data Bank (PDB) [8,9] as a PDB file.

#### Use of UCSF Chimera for Docking the Target Protein

UCSF Chimera is an extensible program that is meant mainly for visualization and analysis of the molecular structures. However, in this paper, we are operating Autodock Vina in Chimera for docking purposes.

**Figure 1:**
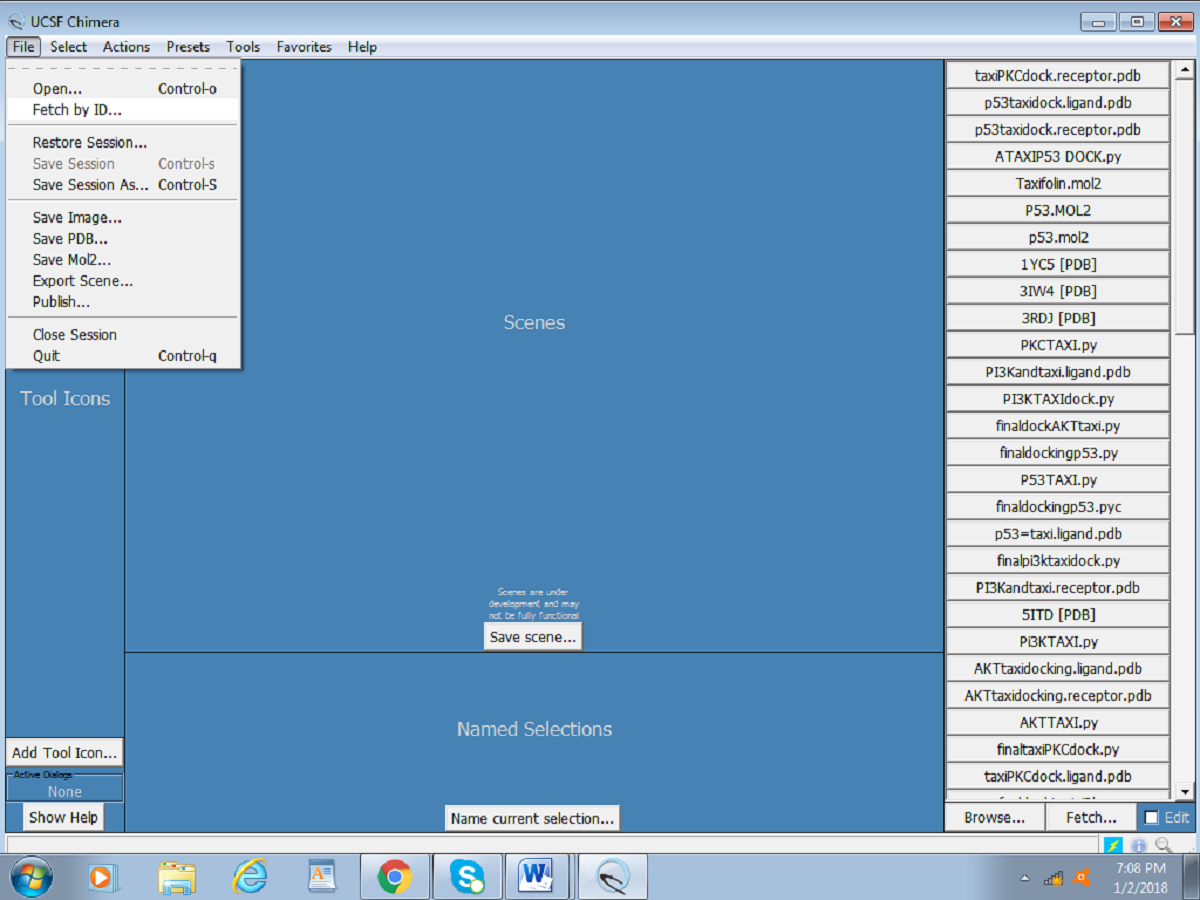
Screenshot of the process to fetch/deliver a protein structure in Chimera.

**Figure 2:**
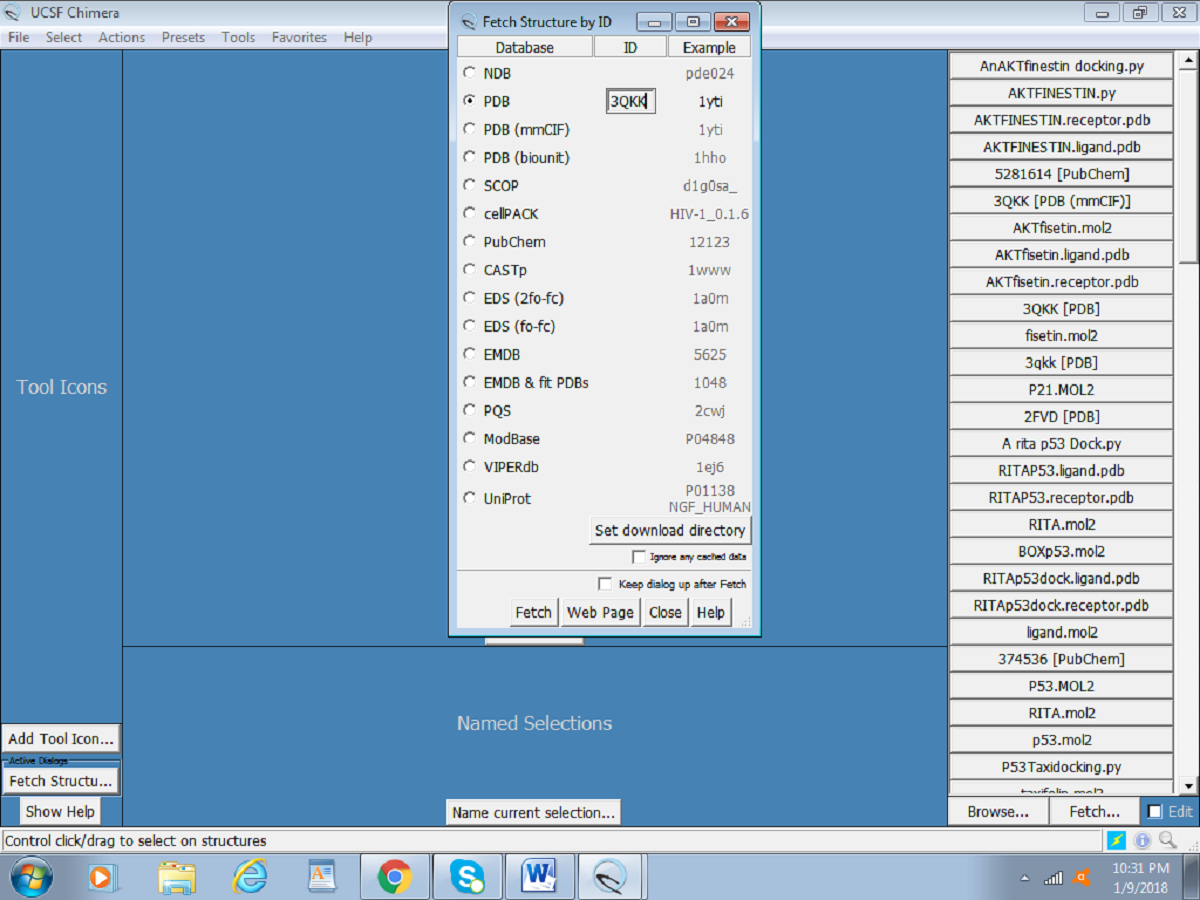
Screenshot of the retrieved protein structure of Akt from the RCSB protein database.

**Figure 3:**
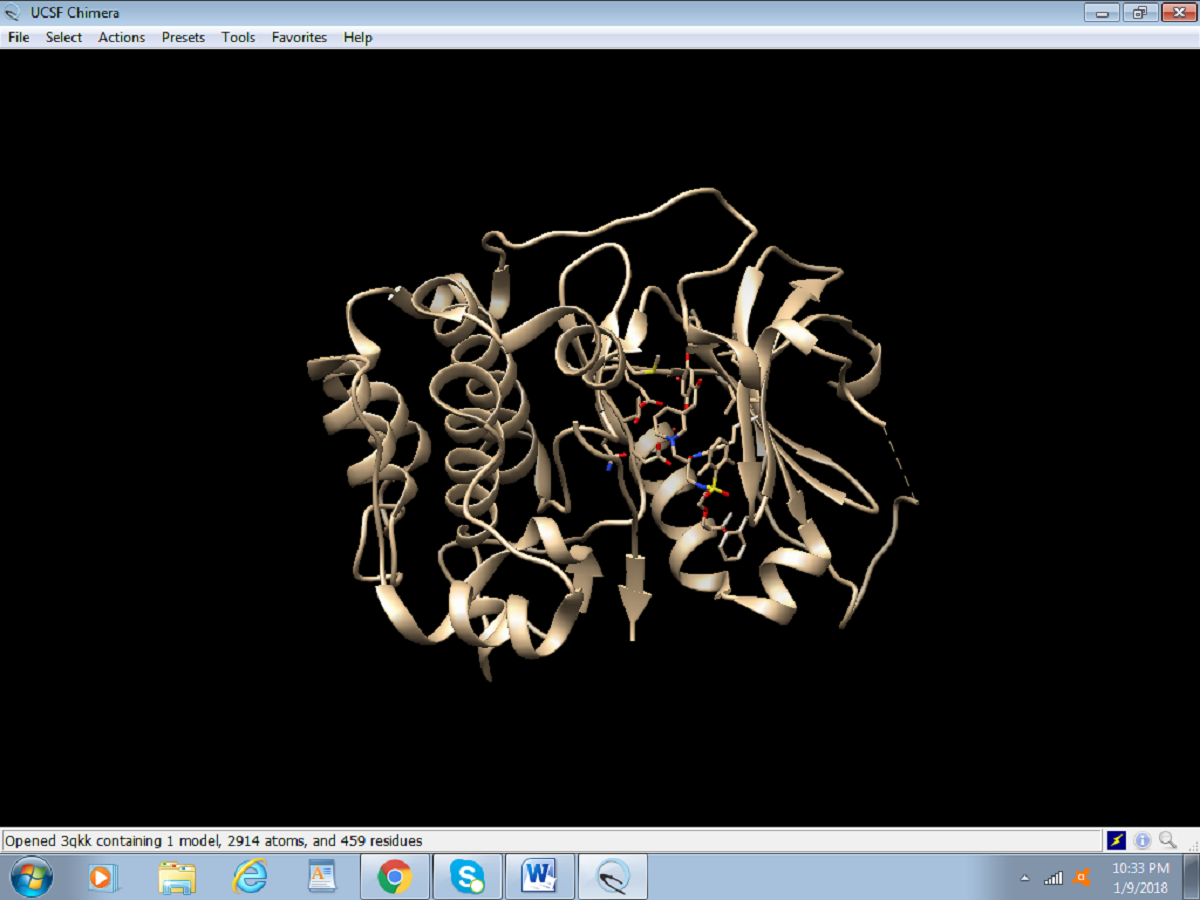
Screenshot of the retrieval of protein database structure of the protein in UCSF (University of California, San Francisco) Chimera.

1. Click on the file and fetch by ID, as shown in Figure 1.
2. Input the PDB ID of the protein (Akt: 3QKK). Figure 2 presents a screenshot of how to obtain the protein structure through PDB ID in Chimera. Any protein can be fetched by inserting the PDB ID of the protein.
3. When the protein is fetched, its structure is downloaded through the website; hence, a working internet connection is required, or the PDB file can be downloaded beforehand and simply be opened thorough File > open. Figure 3 displays the Akt structure retrieved in UCSF Chimera.
4. Create a working directory for the docking project that is convenient to access, such as Users/Desktop/Docking/. Start saving all your prepared files there, for example, save 3QKK as Akt.pdb.

#### Preparing the Target Protein for Docking

**Figure 4:**
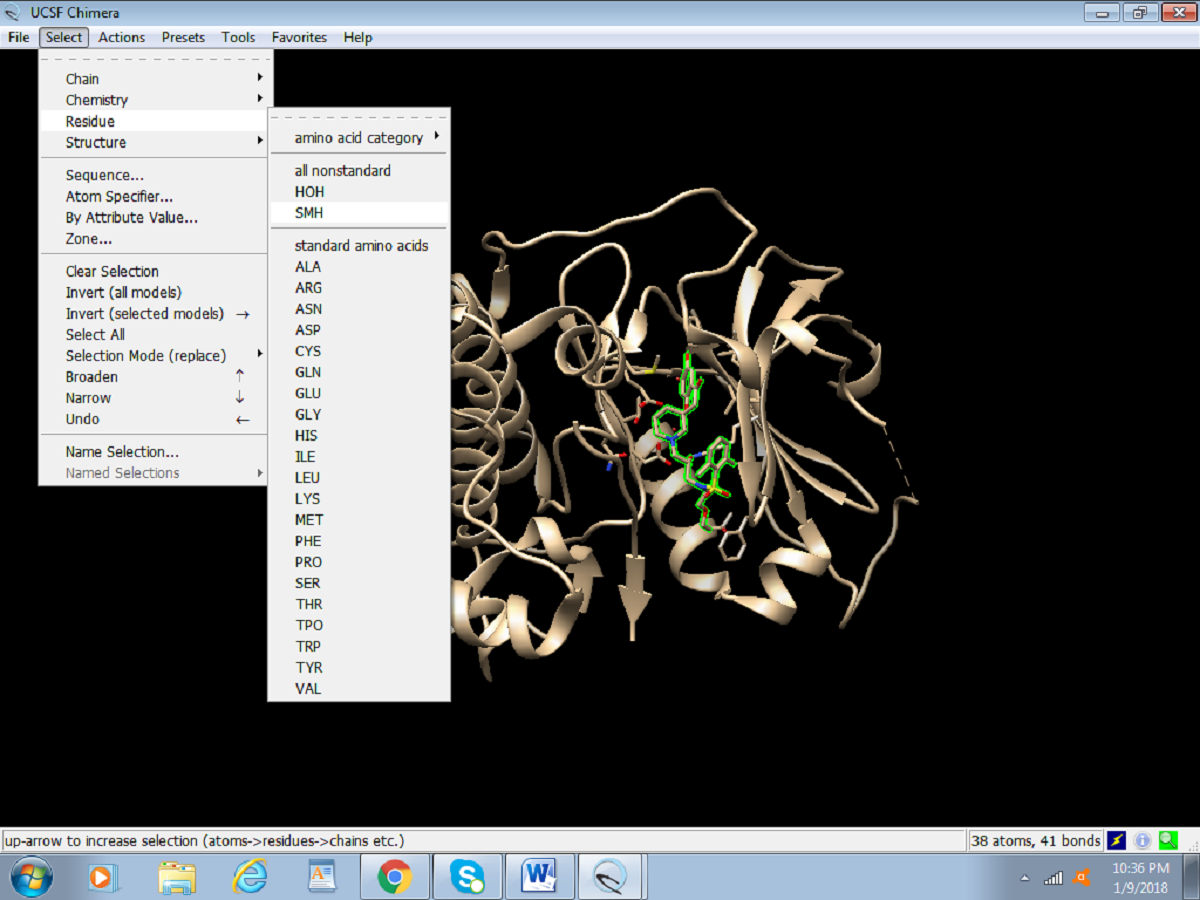
Screenshot of selecting nonstandard residues.

**Figure 5:**
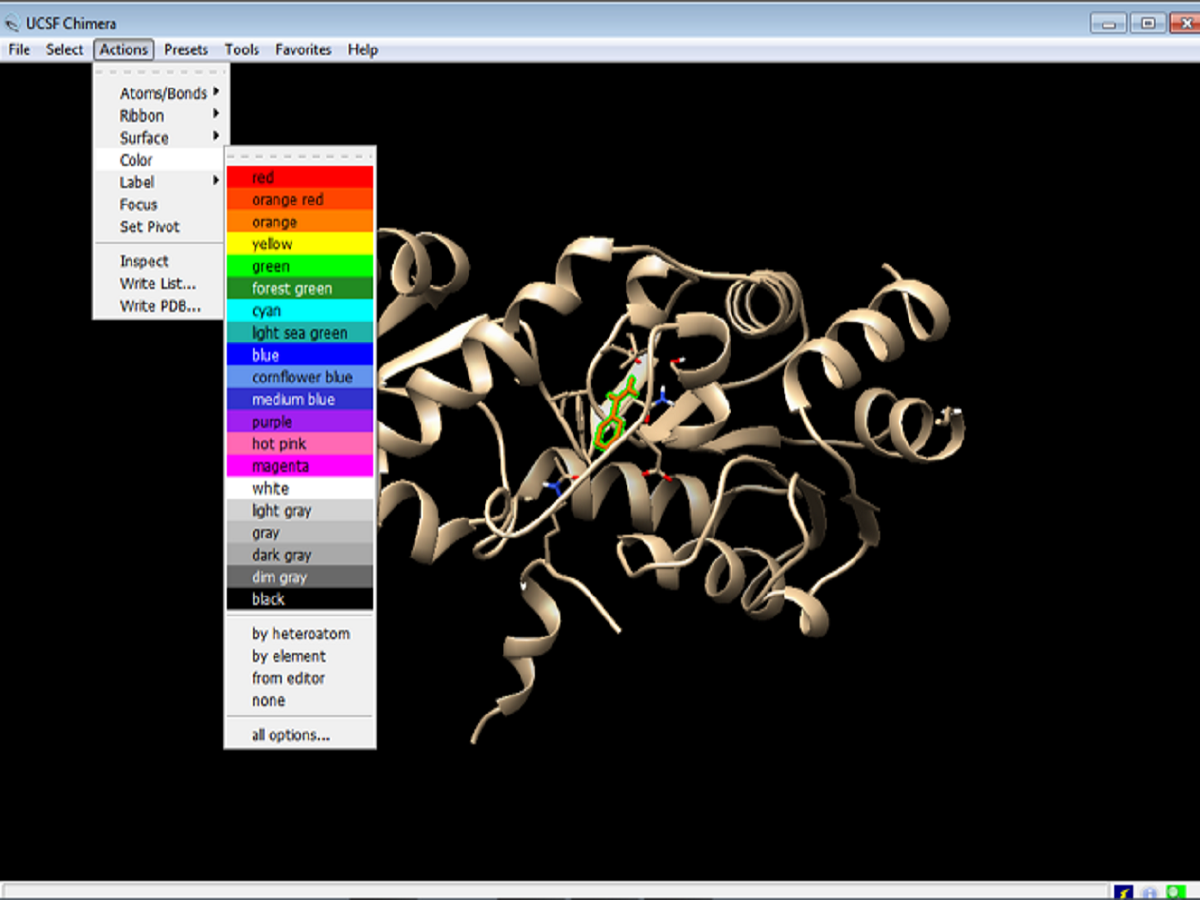
Screenshot of changing the color of the nonstandard (inhibitor) residues.

**Figure 6:**
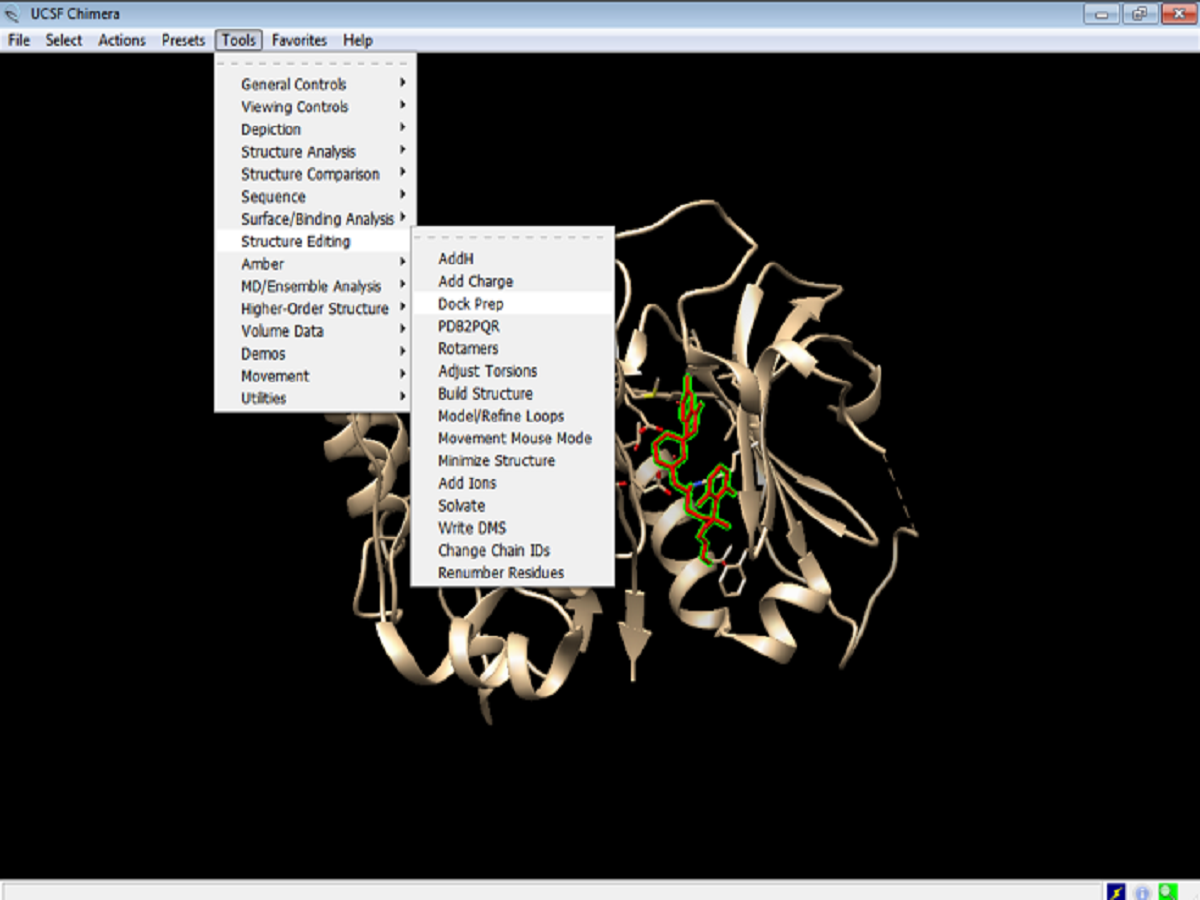
Screenshot of an illustration of preparation of the protein for docking (ie, Dock Prep).

**Figure 7:**
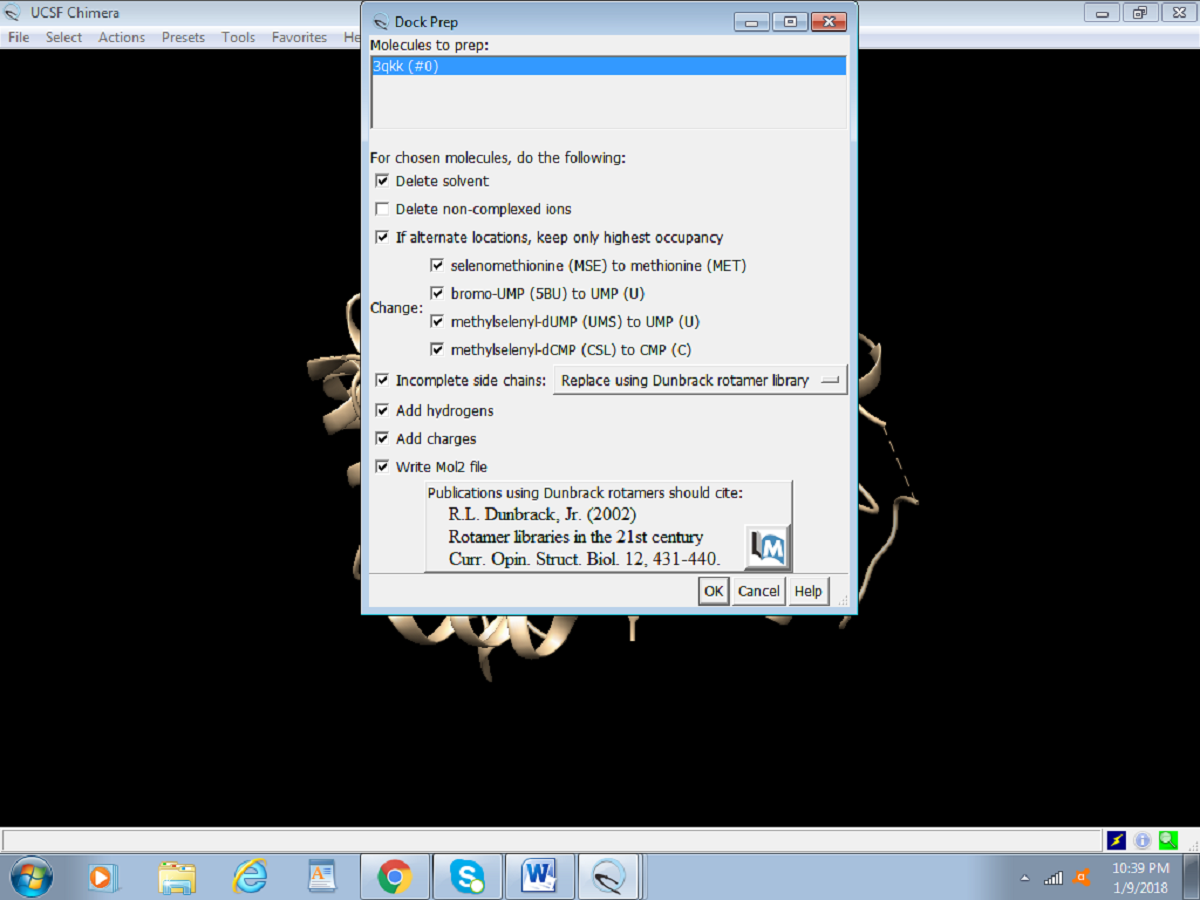
Screenshot of an example of the Dock Prep box that pops up.

**Figure 8:**
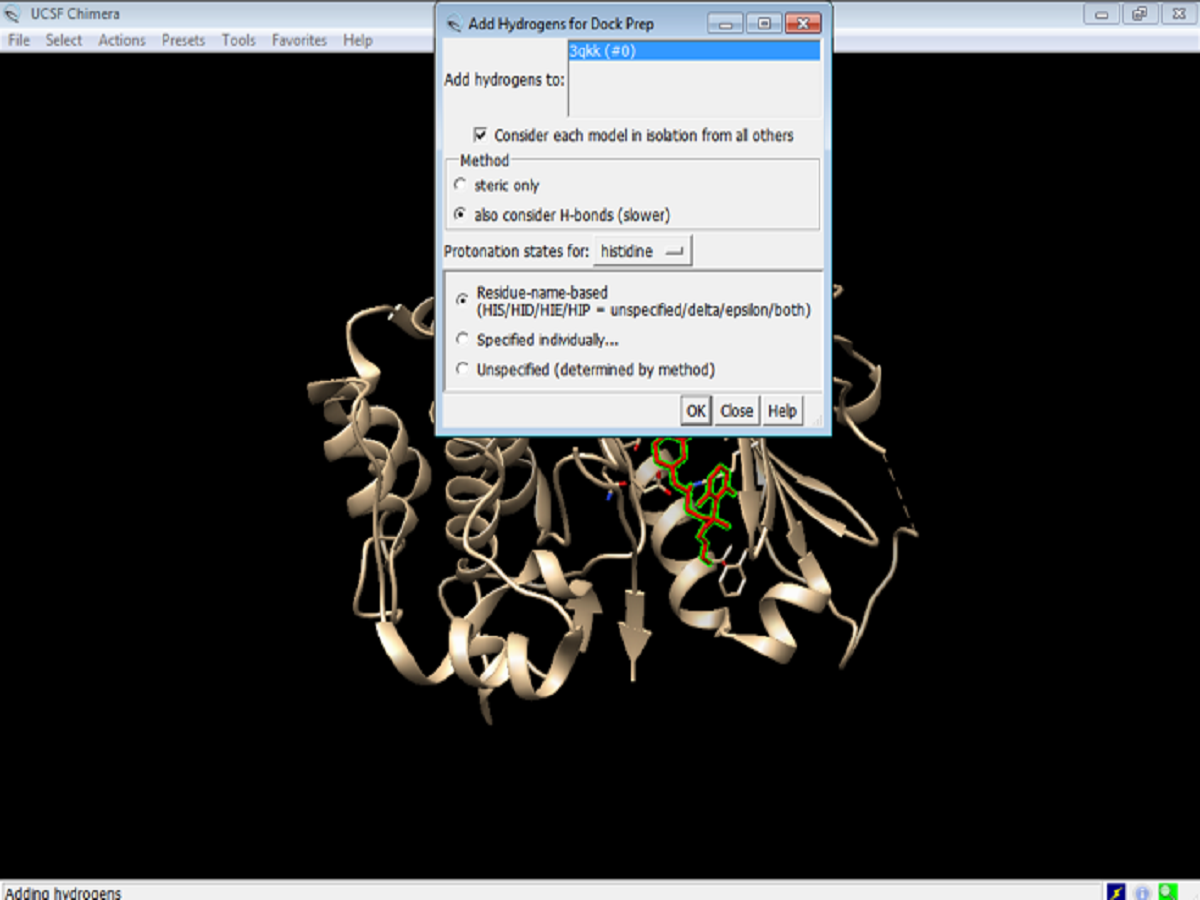
Screenshot of adding hydrogen atoms to the protein.

**Figure 9:**
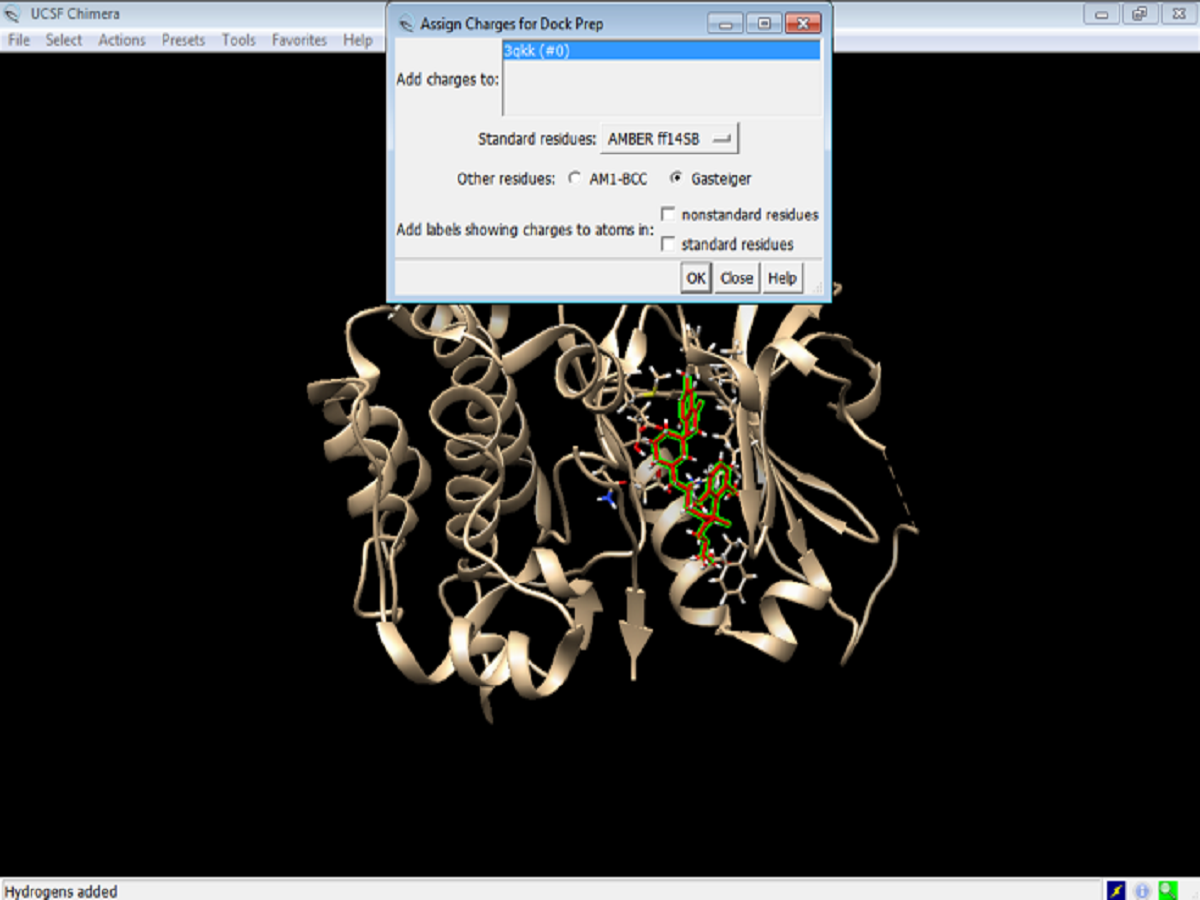
Screenshot of the selection of Gasteiger charges for Akt.

**Figure 10:**
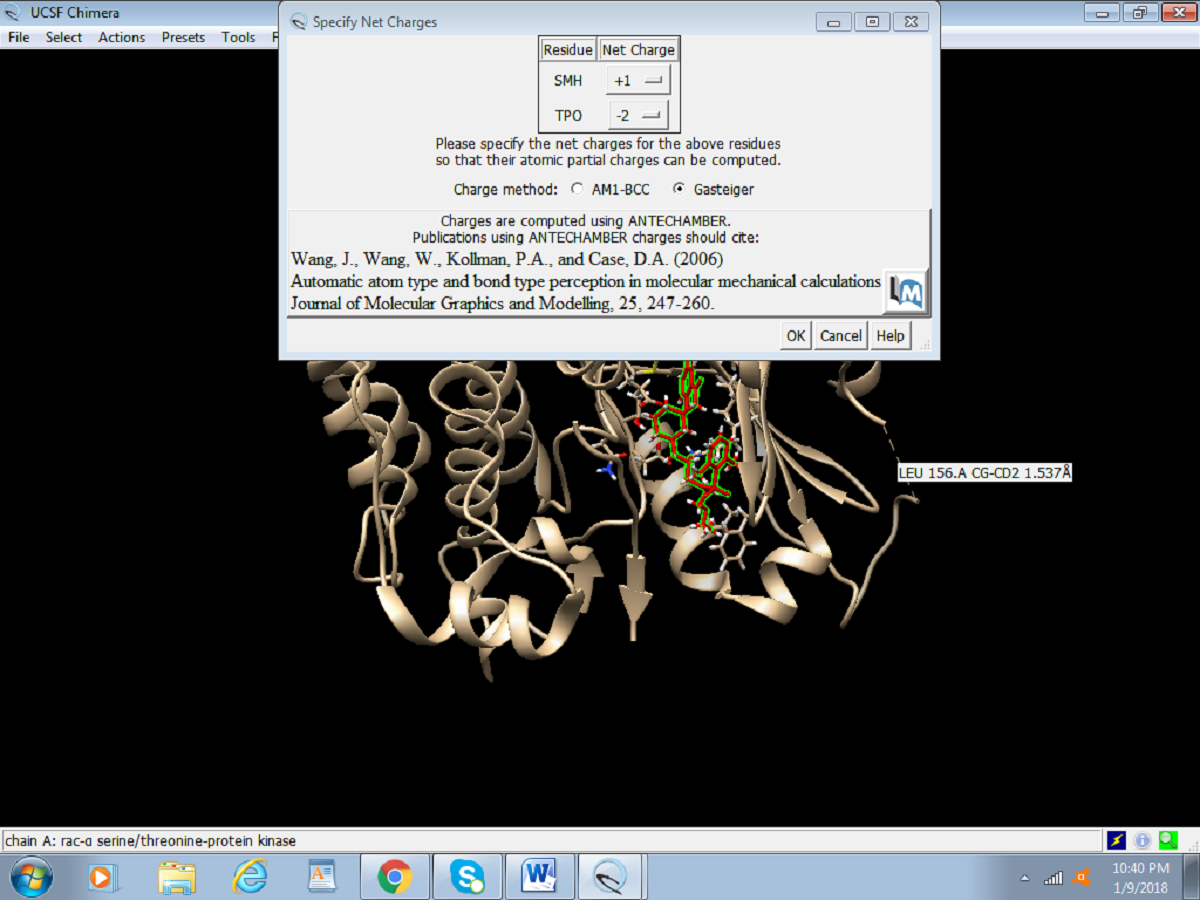
Screenshot of the net charges of a protein.

1. To easily define the active site, the already present inhibitor needs to be identified. To do so, select the inhibitor by click on Select > Residue > SMH (nonstandard residue), as seen in Figure 4. In this screenshot, Akt bears an HOH group and SMH residues as nonstandard residues. Due to the selection, SMH appears to be highlighted in green.

2. After selecting the nonstandard (inhibitor) residues, the residues must be accorded a color. To distinguish the chosen residue from the rest of the protein (Figure 5), change the color by clicking on Actions > Color > red (any color of your choice).

3. The protein needs to be optimized for docking. Click on Tools > Structure Editing > Dock Prep (Figure 6). The required dock prep tools are all available within Chimera. These dock prep tools are available in the structure editing file menu option.

4. In the dock prep box, select all options except “Delete non-complexed ions” and click OK (Figure 7).

5. Add hydrogen to the proteins by selecting the appropriate following options and click OK (Figure 8). We allow the program to make the best choice according to the model by selecting the abovementioned options.

6. Assign charges to the protein by clicking on the Gasteiger charges (Figure 9) and click OK.

7. Select the net charges (Figure 10) and click OK. For most proteins, the net charges equal to zero.

8. Save this file again as preped_Akt.PDB.

#### Preparing the Ligand for Docking

Similar to the process of obtaining the protein, drugs with Pubchem compound ID (CID) can be fetched through the software with a working internet connection.

**Figure 11:**
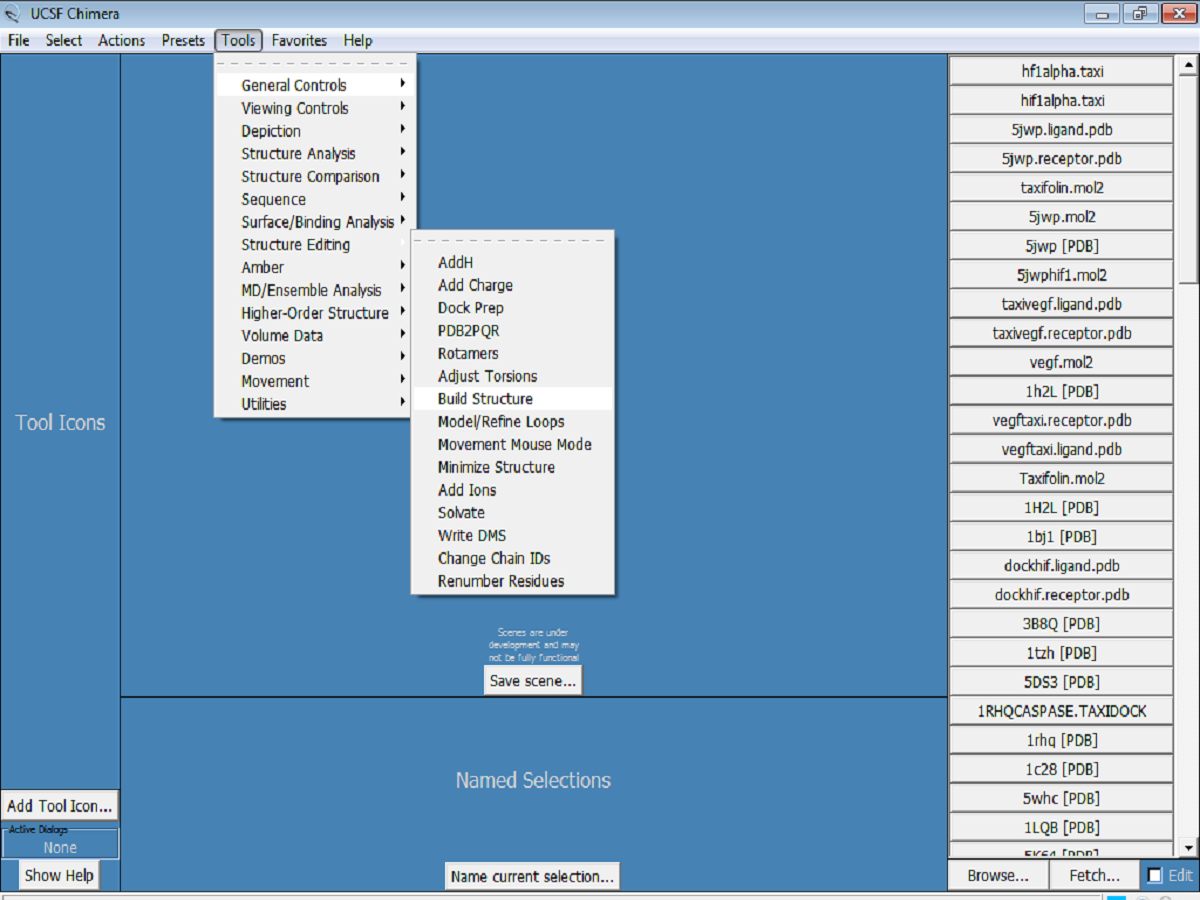
Screenshot of fetching the ligand compound fisetin through its PubChem ID in Chimera.

**Figure 12:**
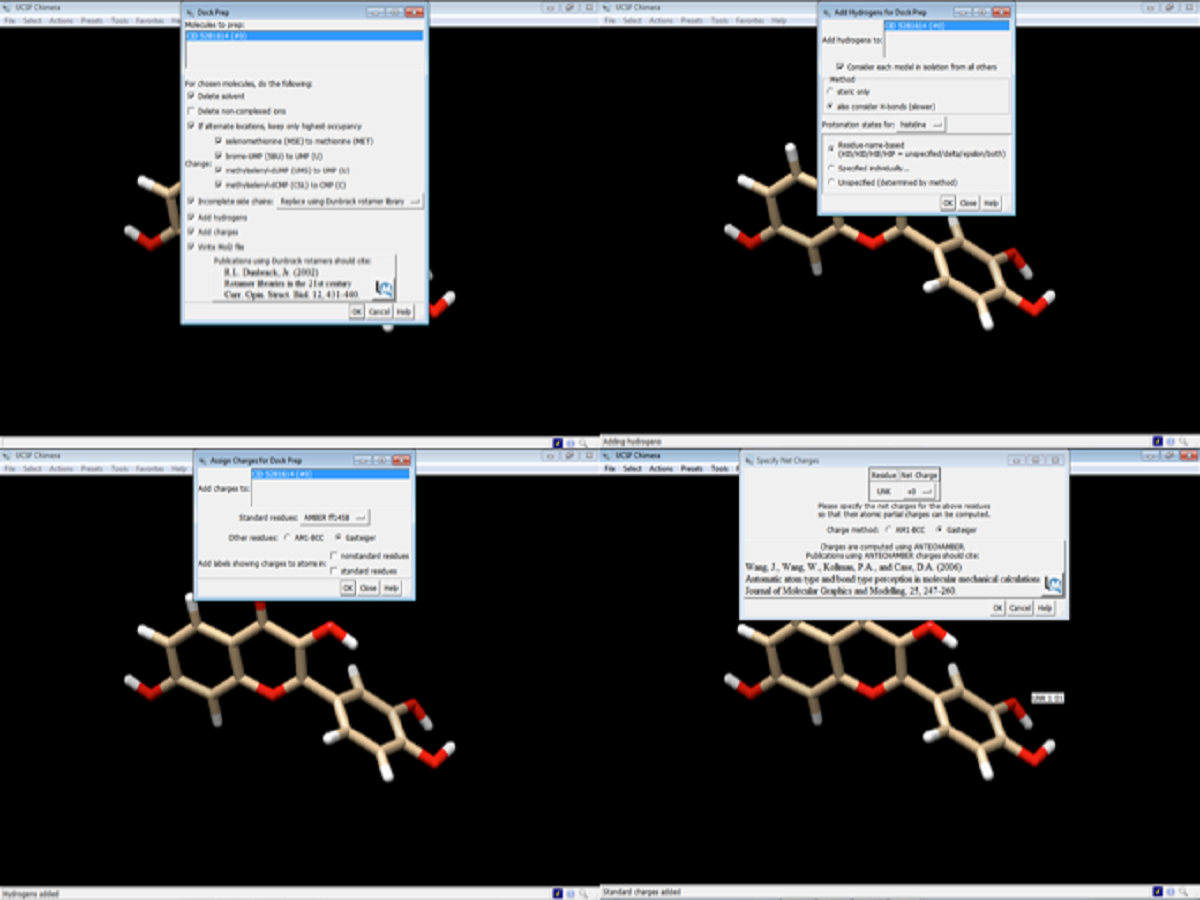
Screenshot of preparing the ligand for docking.

**Figure 13:**
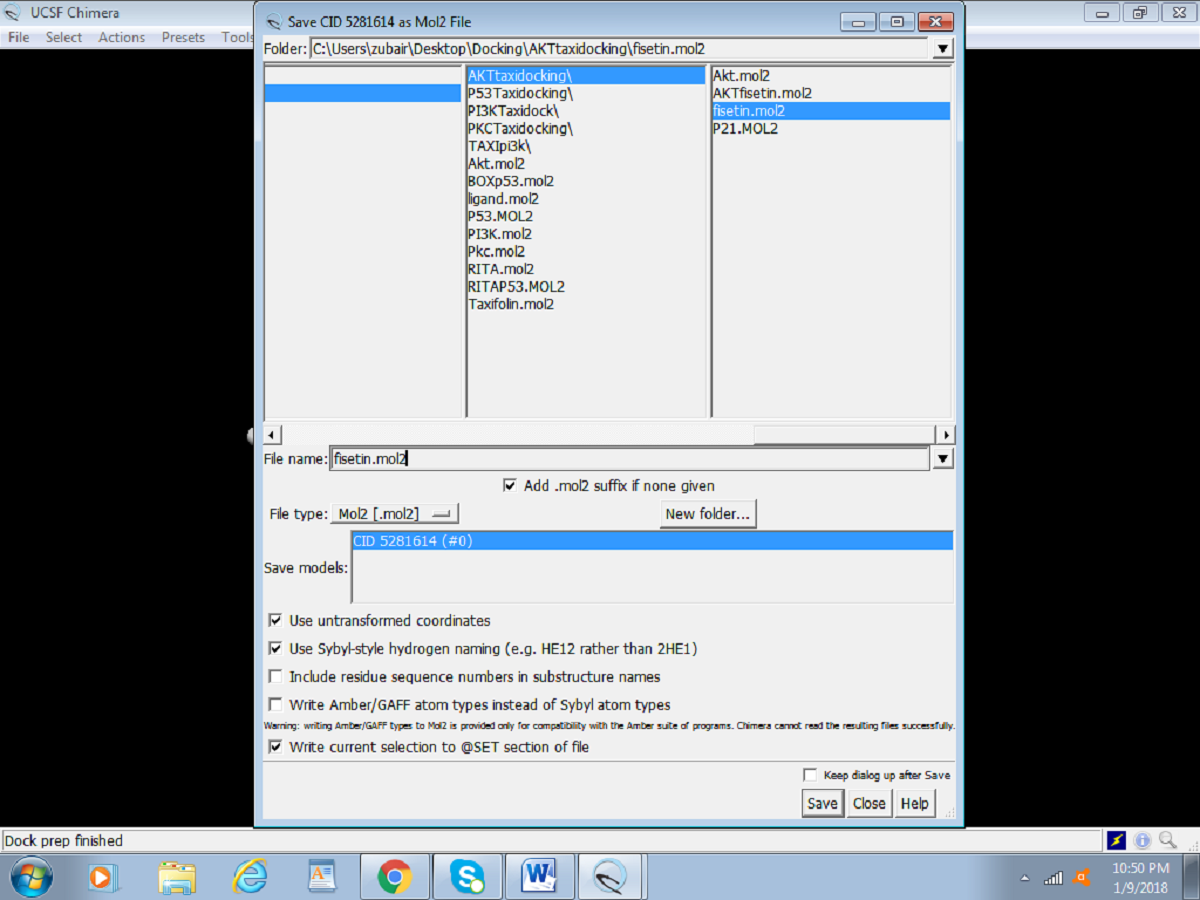
Screenshot illustrating the location of the ligand Mol2 file.

1. Click on Structure Editing > Build Structure > PubChem CID or you can even insert the simplified molecular-input line-entry system (SMILES) of the novel compound being used. Figure 11 shows how to fetch ligands from PubChem using its ID.
2. Enter the PubChem CID and click apply.
3. The ligand needs to be optimized as the protein was optimized. Click on Tools > Structure Editing > Dock Prep, and repeat the same steps followed for preparing the protein. These steps include removing solvents, adding hydrogens, and determining the charge. Figure 12 shows an overview of the dock prep for the ligand.
4. The ligand Fisetin is saved as prep_fisetin.mol2 file in the working directory earlier created (Figure 13).

#### Docking

**Figure 14:**
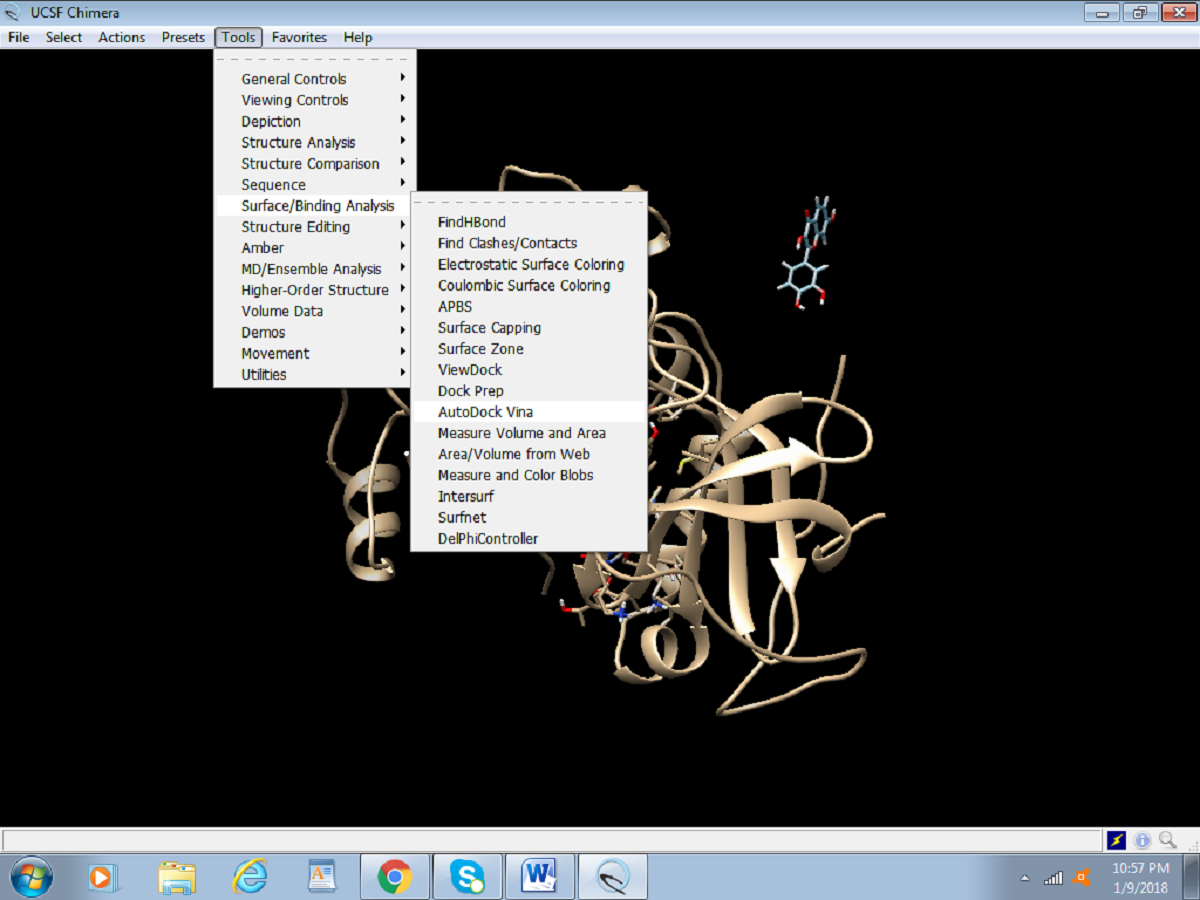
Screenshot of the process to access the Autodock Vina tool in Chimera.

**Figure 15:**
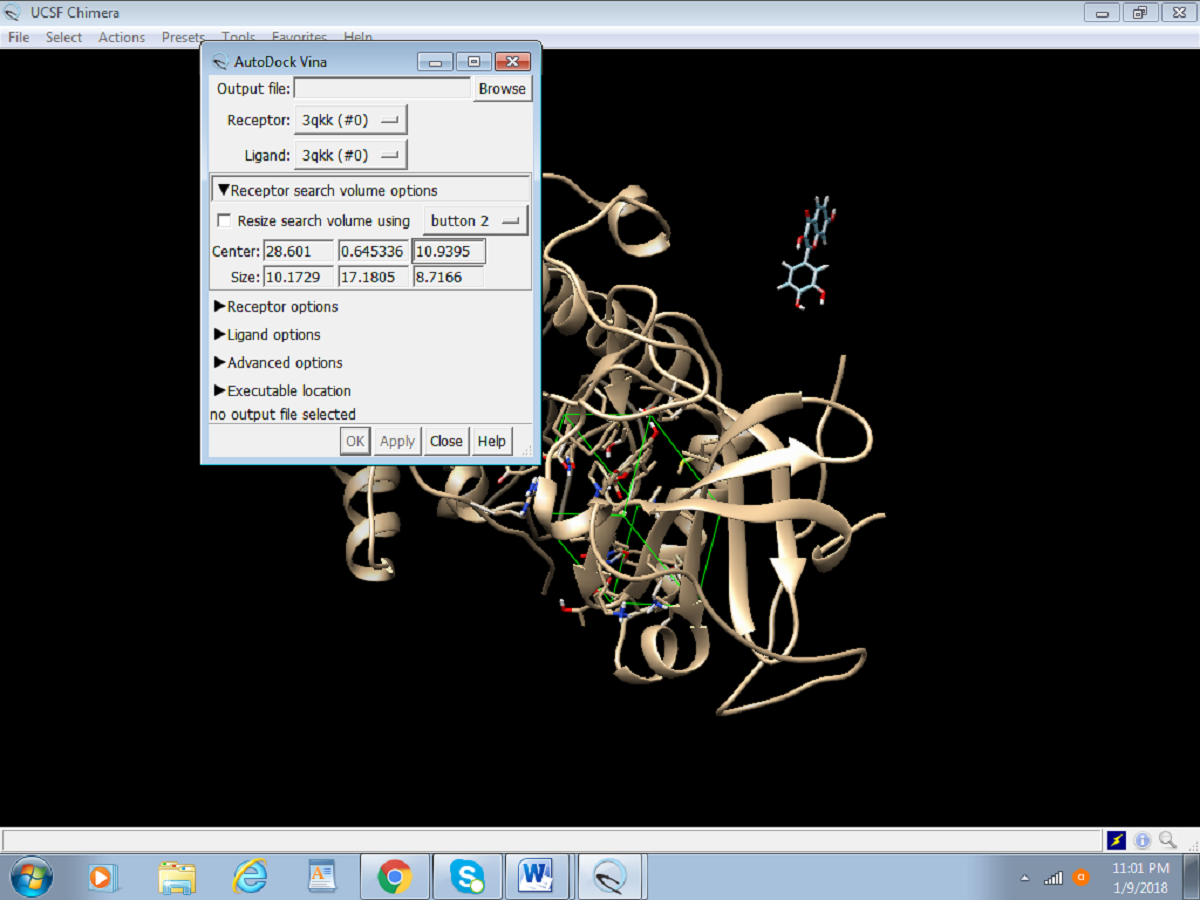
Screenshot of configuring the grid box values in Chimera.

**Figure 16:**
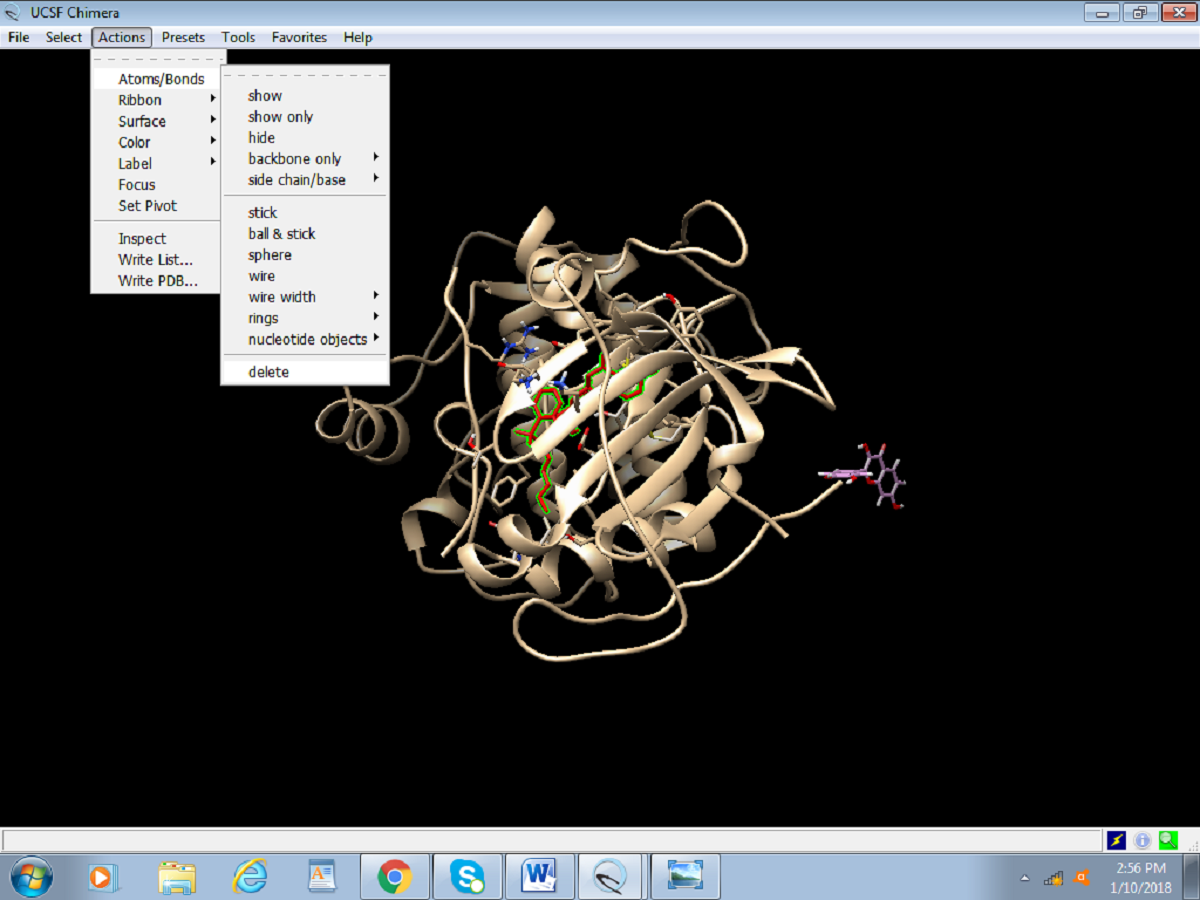
Screenshot of deletion of the inhibitor that is bound to the protein.

**Figure 17:**
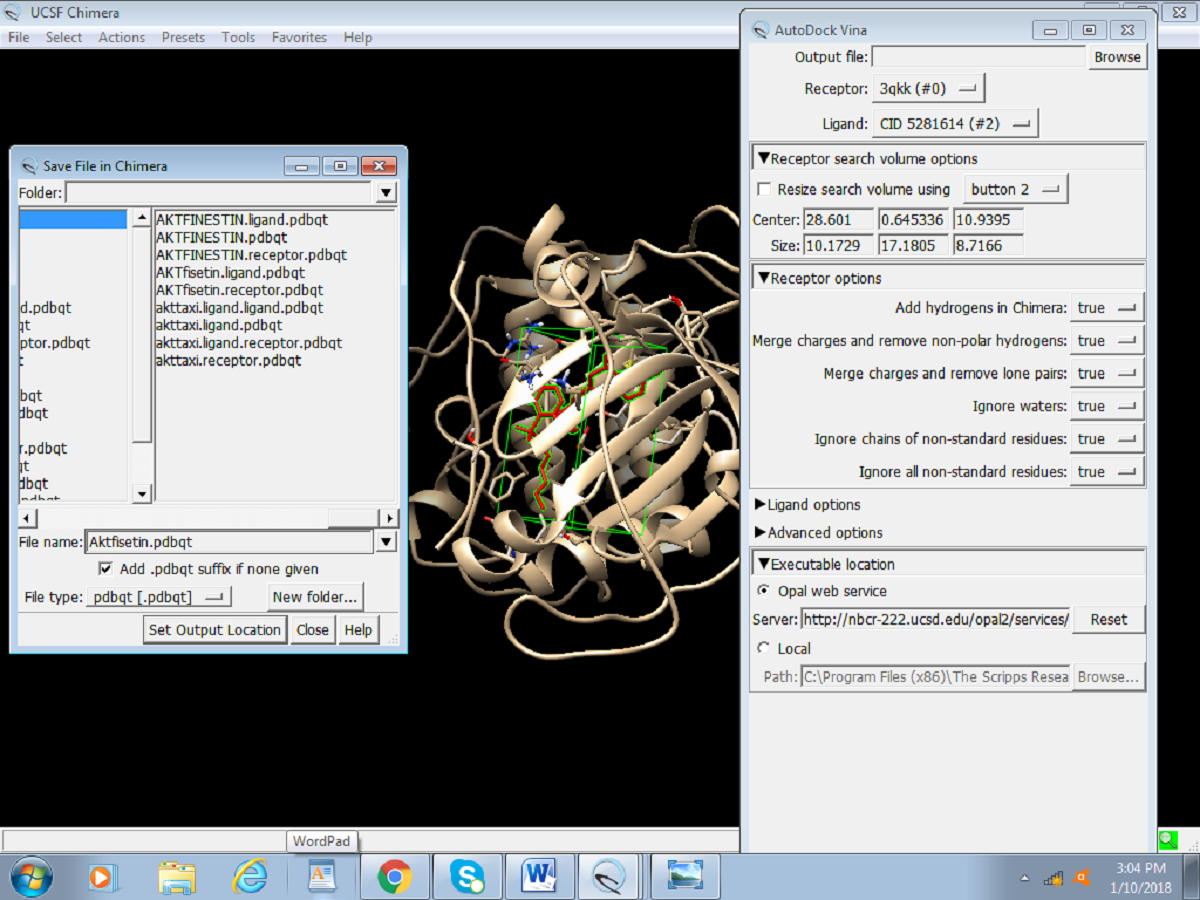
Screenshot of the receptor and ligand options configuration in Autodock Vina.

**Figure 18:**
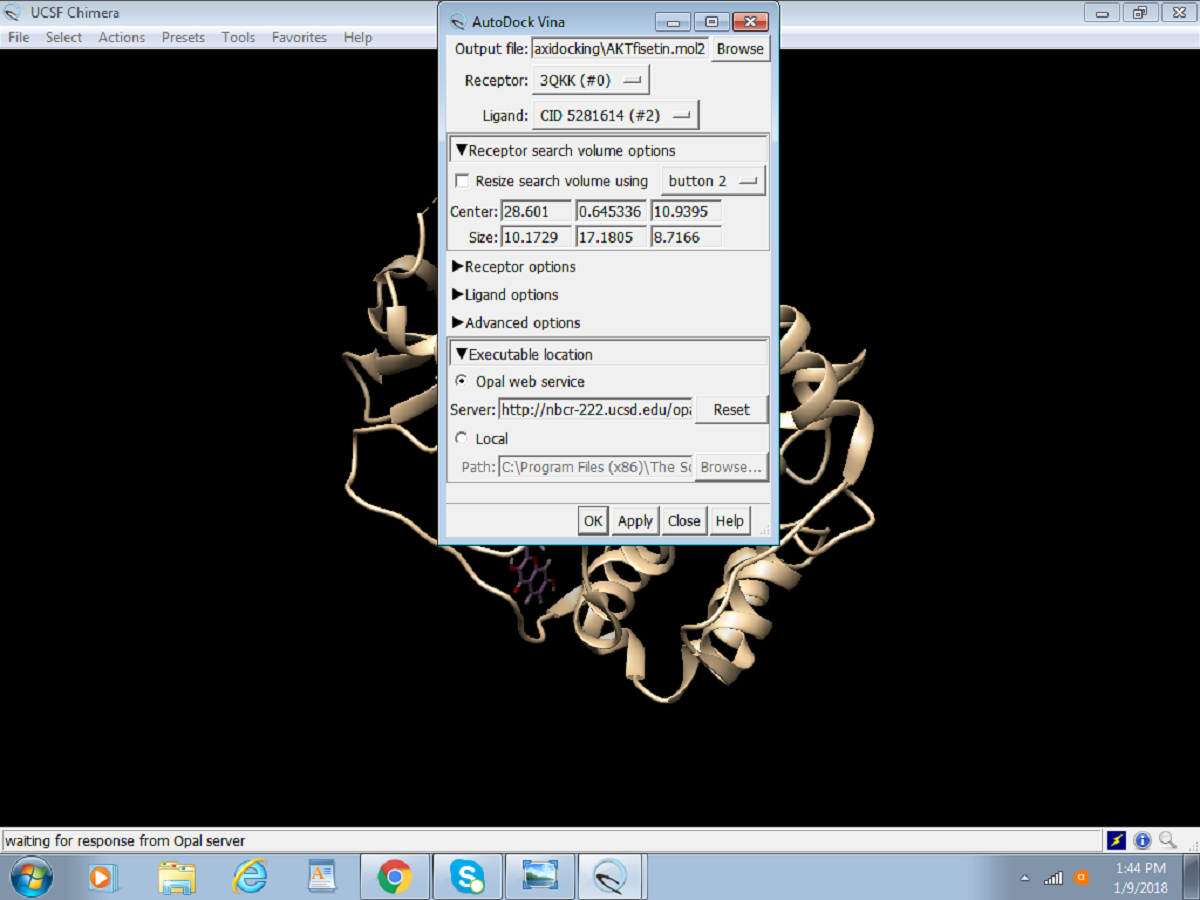
Selection of the Opal web service app in Chimera.

The following steps outline the process for docking:

1. Click on Tools > Surface or Binding Analysis > Autodock Vina (Figure 14).

2. We will set up the grid box values on the active site; this is usually where the previous inhibitor was present. In case an inhibitor is absent or the active site is relatively unknown, the size of the box and the location of the amino acids are determined by reading the literature (Figure 15). For the purpose of this protocol, we will use the active site that already had an inhibitor attached to it.

3. Browse the output file and save as Akt Fisetin.pdbqt in the same directory.

4. Delete the inhibitor molecule attached to the original 3D structure. Thereafter, select Actions > Atoms and Bonds > Delete (Figure 16). The removal of the inhibitor is important to easily visualize the docking results. The 3QKK PDB needs to be saved again as preped_Akt.PDB.

5. Choose the receptor as the protein (preped_Akt) from the drop-down menu and the ligand as prep_fisetin.mol2. It is important to set the right receptor and ligand. In the receptor and ligand options, change everything to TRUE (Figure 17).

6. Select the Opal Web service or enter the local path where the installed version of Autodock Vina is placed and click on OK (Figure 18).

## Results

### Outcome of Docking

**Figure 19:**
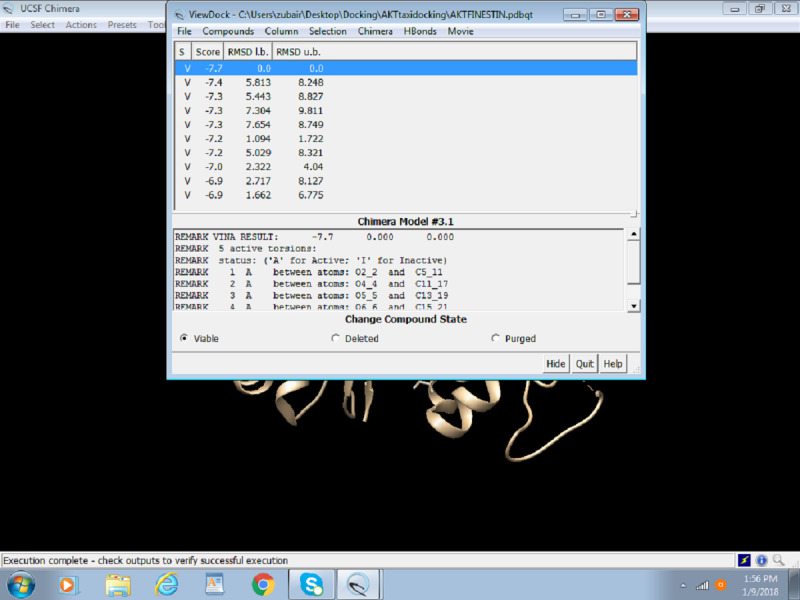
Screenshot of the Result Box after completion of docking.

After the successful run of Autodock Vina, the following dialogue box will appear with the solution. Figure 19 portrays the final step of Docking, that is, outcome/results of docking, which are score, root-mean-square deviation (RMSD) lower bound, and RMSD upper bound.

### Visualizing the Docking

**Figure 20:**
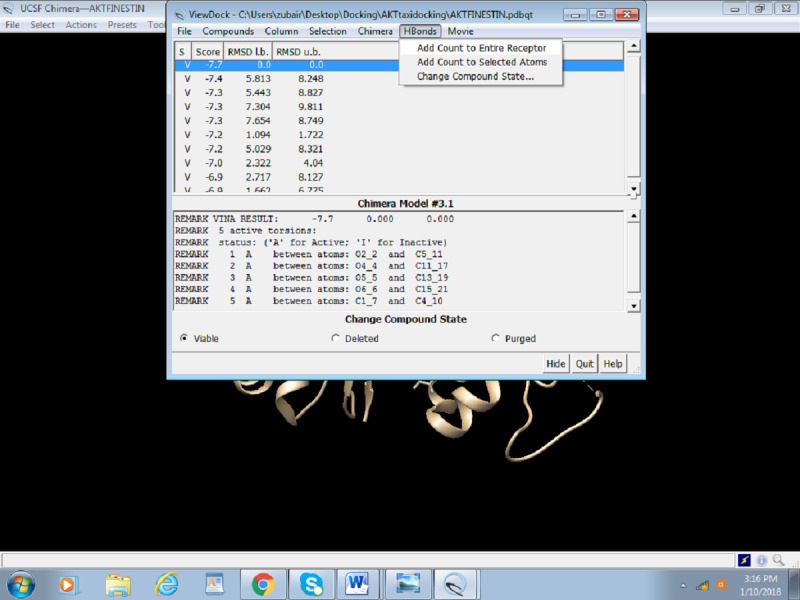
Screenshot of visualizing hydrogen bonding between the receptor and ligand.

**Figure 21:**
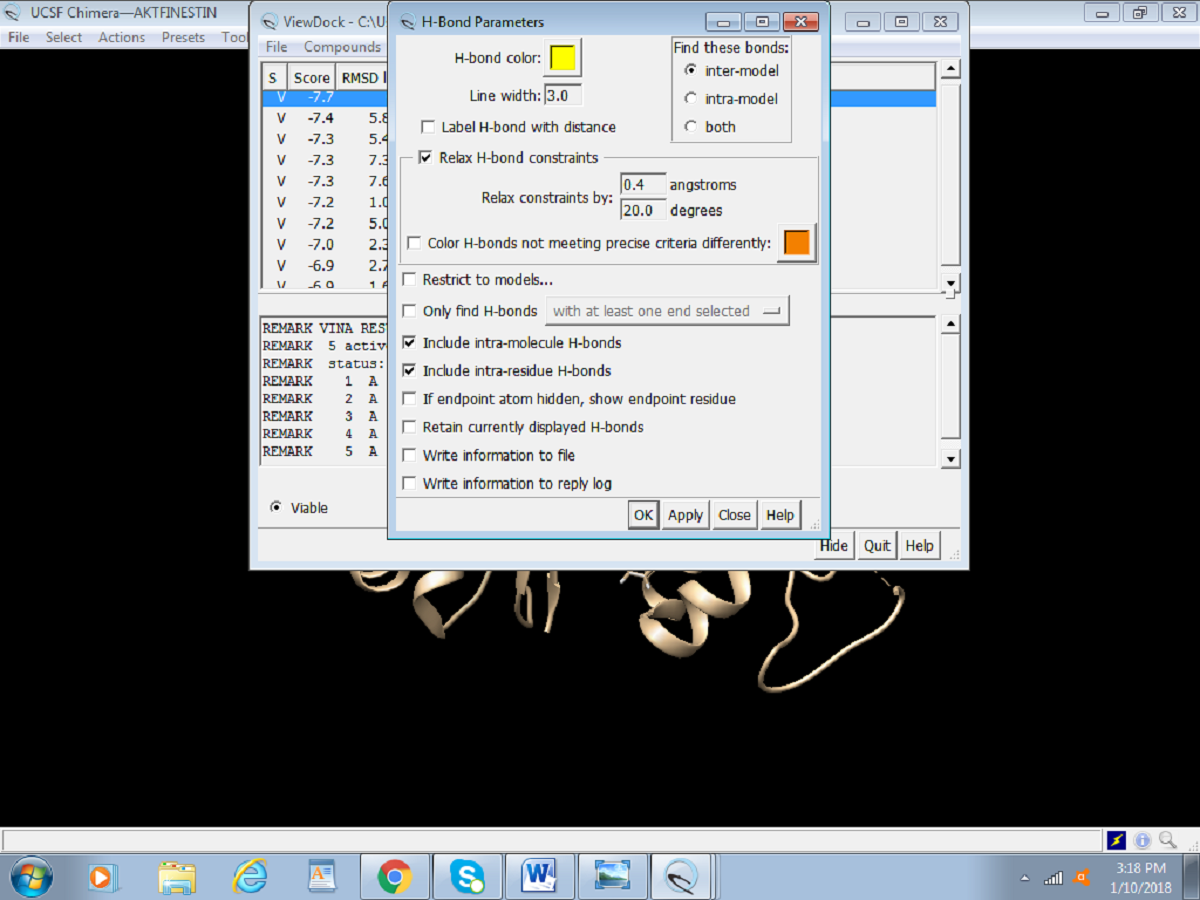
Screenshot of the H-bond Parameters dialogue box.

**Figure 22:**
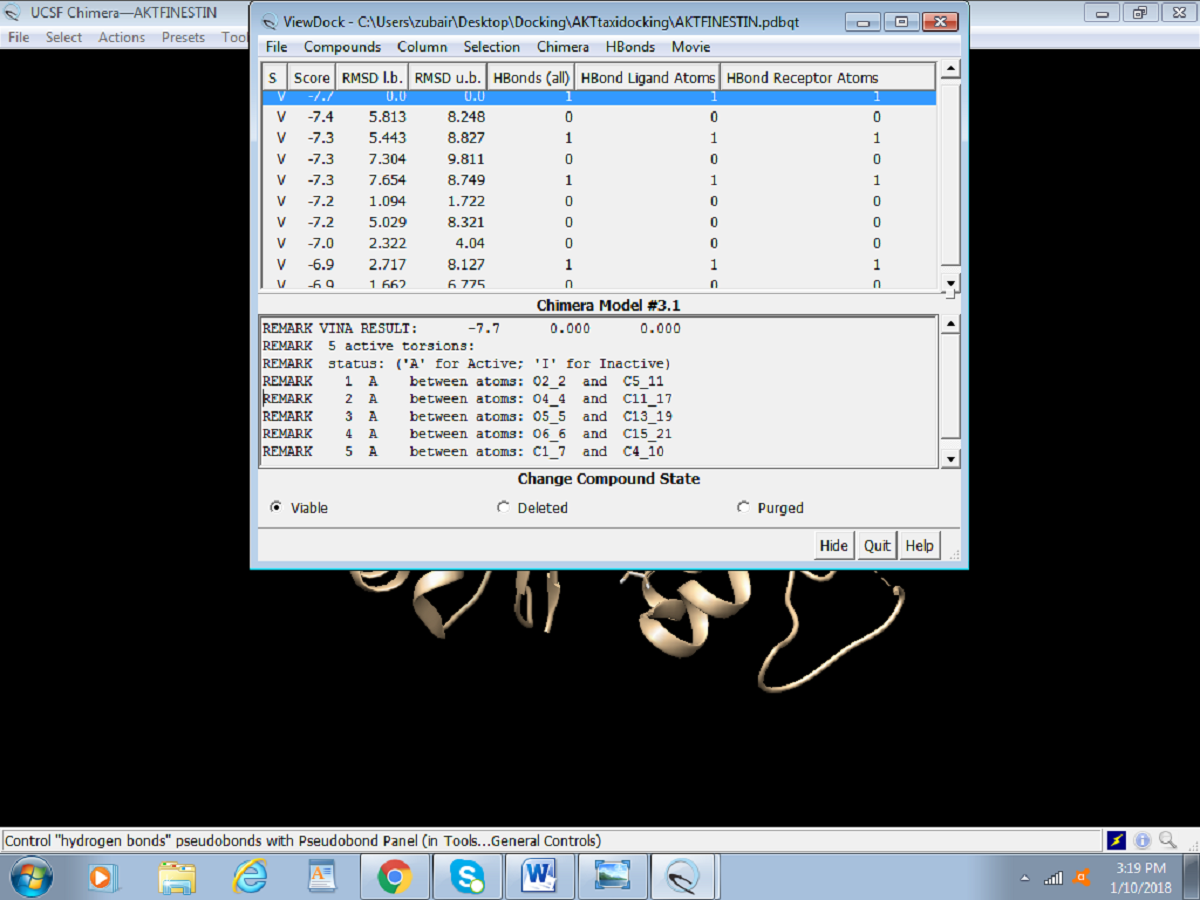
Screenshot of the table showing the number of hydrogen bonds and root-mean-square deviation values.

Visualization of docking can be done as follows:

1. To see the hydrogen bonding between the receptor and the ligands using the result dialogue box (Figure 20), select H Bonds > Add Count to the Entire Receptor.

2. This opens an H-bond parameter dialogue box (Figure 21). Select Intermodel to visualize bonding between receptor and ligand. Different parameters can be adjusted to better picture the bonding. The table showing all the information on hydrogen bonds and RMSD is presented at the end of the docking session (Figure 22). 

3. To be able to retrieve the docking session later at any stage, it can be saved by selecting File > Save Session as > An Akt Fisetin Docking (name the session).

## Discussion

Computationally fast and accurate docking of a ligand with a target protein can be performed using AutoDock Vina in Chimera. This protocol will help researchers who are not able to use Autodock and Autodock Vina due to its command-line interface and do not have access to high-end software such as Gold Suite and Molecular Operating Environment to perform computational docking easily. The use of Chimera with Autodock Vina has not been demonstrated before, and due to the ease of the graphical user interface of Chimera, it can be a go-to tool for someone who is just starting to learn bioinformatics.

## Abbreviations

CID: compound ID
PDB: Protein Data Bank
RMSD: root-mean-square deviation
SMILES: simplified molecular-input line-entry system
UCSF: University of California, San Francisco
